# High-Throughput Sequencing Approach Uncovers the miRNome of Peritoneal Endometriotic Lesions and Adjacent Healthy Tissues

**DOI:** 10.1371/journal.pone.0112630

**Published:** 2014-11-11

**Authors:** Merli Saare, Kadri Rekker, Triin Laisk-Podar, Deniss Sõritsa, Anne Mari Roost, Jaak Simm, Agne Velthut-Meikas, Külli Samuel, Tauno Metsalu, Helle Karro, Andrei Sõritsa, Andres Salumets, Maire Peters

**Affiliations:** 1 Competence Centre on Reproductive Medicine and Biology, Tartu, Estonia; 2 Department of Obstetrics and Gynecology, University of Tartu, Tartu, Estonia; 3 Institute of Bio- and Translational Medicine, University of Tartu, Tartu, Estonia; 4 Tartu University Hospital's Women's Clinic, Tartu, Estonia; 5 Elite Clinic, Sangla 63, Tartu, Estonia; 6 Department of Electrical Engineering (ESAT), STADIUS Center for Dynamical Systems, Signal Processing and Data Analytics, KU Leuven, Leuven, Belgium; 7 iMinds Medical IT, Leuven, Belgium; 8 Centre for Biology of Integrated Systems, Tallinn University of Technology, Tallinn, Estonia; 9 Institute of Computer Science, University of Tartu, Tartu, Estonia; Baylor College of Medicine, United States of America

## Abstract

Accumulating data have shown the involvement of microRNAs (miRNAs) in endometriosis pathogenesis. In this study, we used a novel approach to determine the endometriotic lesion-specific miRNAs by high-throughput small RNA sequencing of paired samples of peritoneal endometriotic lesions and matched healthy surrounding tissues together with eutopic endometria of the same patients. We found five miRNAs specific to epithelial cells – miR-34c, miR-449a, miR-200a, miR-200b and miR-141 showing significantly higher expression in peritoneal endometriotic lesions compared to healthy peritoneal tissues. We also determined the expression levels of miR-200 family target genes E-cadherin, ZEB1 and ZEB2 and found that the expression level of E-cadherin was significantly higher in endometriotic lesions compared to healthy tissues. Further evaluation verified that studied miRNAs could be used as diagnostic markers for confirming the presence of endometrial cells in endometriotic lesion biopsy samples. Furthermore, we demonstrated that the miRNA profile of peritoneal endometriotic lesion biopsies is largely masked by the surrounding peritoneal tissue, challenging the discovery of an accurate lesion-specific miRNA profile. Taken together, our findings indicate that only particular miRNAs with a significantly higher expression in endometriotic cells can be detected from lesion biopsies, and can serve as diagnostic markers for endometriosis.

## Introduction

microRNAs (miRNAs) are small (typically 22 nucleotides in size) non-coding regulatory RNA molecules, which modulate the stability of specific mRNA targets. Therefore changes in miRNA expression that affect target mRNA degradation and/or translation may cause alterations in the dynamic balance between miRNAs and their target mRNAs, and lead to pathological changes. An altered miRNA expression profile has been associated with uterine and endometrial disorders such as uterine leiomyoma [1], endometrial carcinoma [2] and endometriosis (reviewed [3]).

Endometriosis is one of the most studied gynaecological diseases, but regardless of extensive studies, the pathogenesis of the disease has still remained obscure. A number of studies on eutopic or ectopic endometria have suggested the involvement of miRNAs in endometriosis development, and distinct miRNA expression profiles of eutopic and/or ectopic endometrium from women with endometriosis have been described [4], [5], [6], [7], [8], [9], [10], [11], [12], [13]. Many dysregulated miRNAs have been identified but only a small subset of miRNAs has been repeatedly recognized as disease-related. However, the down-regulation of miR-200 family members in endometriotic lesions compared to eutopic endometria has been shown in three different studies [4], [5], [14]. The miR-200 family regulates two essentially important biological processes: cell migration and epithelial-mesenchymal transition (EMT) that are both supposed to be crucial events in the development of endometriosis [15].

The results of endometriosis miRNA studies have provided great knowledge about the local miRNA expression in eutopic or ectopic tissue but as most of abovementioned studies have focused only on specific predefined subsets of miRNAs analysed by real-time PCR or microarrays, the full miRNome of eutopic or ectopic endometrium of endometriosis patients is still a field that needs to be explored. Next generation sequencing not only measures the absolute number of miRNA abundance, but also enables to find novel miRNAs, therefore offering new possibilities for describing the miRNome of endometriotic lesions and endometrium. To date, only one study has used high-throughput sequencing for profiling the miRNome of endometrioma and found many up- and down-regulated miRNAs in endometriomas compared to eutopic endometria [14]. However, the full miRNome of peritoneal lesions is still unstudied.

The most widely used approach in endometriosis miRNA expression studies is to compare the miRNA profile of ectopic endometrium to that of the eutopic endometrium. However, biopsied endometriotic lesions usually contain only a small proportion of endometrial glands and stroma, and a larger proportion of surrounding tissue with its own miRNA expression pattern, which may mask the disease-specific miRNA expression profile. Therefore, the purpose of this study was to use high-throughput sequencing to explore the endometriotic lesion-specific miRNA expression profile by comparing a set of paired samples of peritoneal endometriotic lesions and matched healthy surrounding tissues together with eutopic endometrium of the same patients.

## Materials and Methods

### Ethics statement

The study was approved by the Research Ethics Committee of the University of Tartu (Tartu, Estonia) and written informed consent was obtained from all participants.

### Study Subjects and Tissue Processing

Eleven tissue samples (two endometria, five peritoneal lesions and four matched adjacent normal-appearing tissues) from two patients with a histologically confirmed diagnosis of moderate-severe endometriosis (III–IV stage) undergoing laparoscopy at the Elite Clinic (Tartu, Estonia) were used for sequencing analysis. The severity of endometriosis was classified according to the American Society for Reproductive Medicine revised classification system [16]. The clinical characteristics as well as menstrual cycle phases of the patients are listed in Table S1.

For validation study additional tissue samples from women undergoing laparoscopy at Tartu University Hospital Women's Clinic and Elite Clinic were included. Together with sequencing study samples the validation set consisted of: 1) histologically confirmed peritoneal endometriotic lesions (n = 22) and 2) non-diseased tissues (altogether 24 samples: 14 samples of adjacent normal-appearing tissues and 10 samples of seemingly endometriotic lesions that were histologically evaluated and confirmed not to be endometriotic lesions). The general characteristics of patients and the list of studied tissues are presented in Table S2. Furthermore, 17 eutopic endometrial biopsies (nine patients with endometriosis and eight healthy women) were collected for endometrium miRNA expression study (Table S3). Additional five endometrial biopsies from healthy women were used to separate endometrial epithelial and stromal cells by fluorescence-activated cell sorting (FACS) (Table S4).

Endometrial biopsy samples were collected using an endometrial suction catheter (Pipelle, Laboratoire CCD, France). Endometriotic lesions and macroscopically healthy surrounding tissues adjacent to the lesions (referred hereafter as ‘healthy tissue’) were removed during laparoscopy. Tissue samples were immediately placed into RNA*later* (Ambion, Inc., Austin, Texas, USA) or cell culture medium. After 24-hour incubation in RNA*later* at 4°C, tissues were stored at −80°C until use.

OCT (Leica, Germany) embedded tissue sections were cut (10 µm), mounted on standard microscope slides, stained with hematoxylin/eosin and evaluated histologically. The sectioning, examination and tissue collection for miRNA extraction was performed as follows: after the trimming, five to ten tissue sections were stained, examined and in case of a negative finding, the following 10–15 slices were collected into a microtube. The sectioning, examination and collection of the tissue was carried on until a positive finding (presence of endometrial epithelial and/or stromal cells) or until all the sample was sectioned through (histologically negative finding). In case of a positive finding, the sectioning was stopped and the remaining biopsy was added to the tube containing previously collected tissue sections.

### Fluorescence-activated cell sorting

Single cell suspensions, obtained from the endometrial biopsies using enzymatic digestion with collagenase (0.5%, Sigma Aldrich, St. Louis, MO), were analysed by FACS using BD FACSCalibur flow cytometer (BD Biosciences, San Jose, CA, USA). Endometrial stromal cells were stained with fluorescence-conjugated rat anti-human CD13 monoclonal antibody (1∶5 dilution, clone 1R3-63, R-Phycoerythrin, Novus Biologicals, Cambridge, UK) and epithelial cells with fluorescence-conjugated mouse anti-human CD9 monoclonal antibody (1∶20 dilution, clone MEM-61, FITC, Novus Biologicals, Cambridge, UK). Target cell populations were sorted directly to QIAzol Lysis Reagent (Qiagen, Hilden, Germany) and total RNA was isolated immediately.

### RNA extraction and sequencing data analysis

Total RNA together with miRNA enriched fraction was extracted using RNeasy MinElute Cleanup kit in combination with miRNeasy Mini kit (Qiagen) according to the manufacturer's instructions (“Preparation of miRNA-Enriched Fractions Separate from Larger RNAs”, appendix A). The quality of total RNA was assessed on RNA 6000 Nano Chip using Agilent 2100 Bioanalyzer and the presence of miRNAs in enriched samples with Agilent Small RNA chips (Agilent Technologies, Palo Alto, CA, USA).

Small RNA library construction and miRNA sequencing were performed at Biomedicum Functional Genomics Unit (Helsinki, Finland) as described previously [17].

The quality of raw miRNA sequencing data was assessed by FastQC program (http://www.bioinformatics.babraham.ac.uk/projects/fastqc/). Filtered sequencing reads were analysed with miRDeep2 software [18] designed to predict known and novel miRNAs from deep sequencing data. Default parameters were used in all analysis steps. Briefly, adapter sequences were trimmed from the raw reads. Reads with at least 18 nucleotides were retained and then aligned to the human genome (NCBI build 37, hg19). Sequencing reads that mapped more than five times to the genome were discarded from the dataset. The remaining reads were used for generating potential miRNA precursors. These sequences were aligned to miRBase release 18 [19] allowing one mismatch to detect previously annotated miRNAs and estimating their expression levels from deep sequencing data. The reads predicted as potential novel miRNAs by miRDeep2 were subjected to BLAST to remove the sequences corresponding to human non-coding RNAs such as rRNA, snRNA, and tRNA. Novel miRNAs were identified by the following criteria: read count five or more and miRDeep score at least 2. Annotated miRNA read counts were normalised to the total read counts of all reads per sample (expressed as counts per million). To test differential miRNA expression between endometriotic lesions, healthy tissues surrounding the lesions and eutopic endometria, expression data for known miRNAs produced by miRDeep2 was used as input for the program R (version 2.15.2) package edgeR [20] available in Bioconductor version 2.8. Differential miRNA expression analysis was performed by edgeR and miRNAs with <5 raw counts in more than half of the samples were discarded. The p-values were adjusted for multiple testing using the Benjamini and Hochberg method. miRNA levels with false discovery rate (FDR) -adjusted p-values<0.1 were considered statistically significant. To explore miRNA expression profile similarities between eutopic endometria, ectopic lesions and healthy tissues, multidimensional scaling (MDS) plots were created using edgeR. All data obtained via sequencing is available at Gene Expression Omnibus data repository (http://www.ncbi.nlm.nih.gov/geo/), accession number GSE56414.

### Validation of miRNA expression by quantitative real-time PCR (qRT-PCR)

Five miRNAs: hsa-miR-449a (Applied Biosystems, Assay ID 001030), hsa-miR-34c-5p (ID 000428), hsa-miR-200a-3p (ID 000502), hsa-miR-200b-3p (ID 002251) and hsa-miR-141-3p (ID 000463) were selected for validation analysis (denoted hereafter as miR-449a, miR-34c, miR-200a, miR-200b, and miR-141). Two small nucleolar RNAs, RNU44 (ID 001094) and RNU48 (ID 001006) showing stable expression in human endometrium [21] were used as endogenous controls. cDNA synthesis was conducted from miRNA enriched RNA fraction with TaqMan MicroRNA Reverse Transcription Kit (Applied Biosystems, California, US). Real-time PCR was performed in 7500 Fast Real-Time PCR System (Applied Biosystems) using TaqMan Universal PCR Master Mix, No AmpErase UNG (Applied Biosystems) and following the manufacturer's instructions.

### E-cadherin, ZEB1 and ZEB2 mRNA expression by qRT-PCR

E-cadherin, ZEB1 and ZEB2 (E-box-binding transcription factors 1 and 2) expression levels were determined in 12 paired endometriotic lesions and adjacent healthy tissue samples (Table S2) using the following primers for: ZEB1 (Fw-GACAGTGTTACCAGGGAGGAGCA; Rev-TTCAGGTGCCTCAGGAAAAATGA) [22]; ZEB2 (Fw-CAAGGAGCAGGTAATCGCAAGT; Rev-GGAACCAGAATGGGAGAAACG) [23] and E-cadherin (Fw-CGGGAATGCAGTTGAGGATC; Rev-AGGATGGTGTAAGCGATGGC) [24]. SDHA (Fw-TGGGAACAAGAGGGCATCTG; Rev-CCACCACTGCATCAAATTCATG) [25] was used as endogenous control. DNase treated (TURBO DNA-free kit, Ambion Inc., Austin, Texas, USA) RNA (up to 1 µg) was converted into cDNA using RevertAid First Strand cDNA Synthesis Kit (Thermo-Fisher Scientific Inc. MA, USA). qRT-PCR was performed using 5×HOT FIREPol EvaGreen qPCR Mix Plus (ROX) master mix (Solis BioDyne, Estonia) according to conditions specified by the manufacturer.

### Statistical analysis

The 2^−ΔΔCt^ method [26] was used for calculating the relative expression and to determine the fold change (FC) of miRNA/mRNA expression between endometriotic lesions and adjacent healthy tissues. Normalized miRNA expression level differences between the endometriotic lesions and healthy tissues were analysed using Student's two sided t-test, and *P*<0.05 was considered as a statistically significant difference. The associations between the expression levels of ZEB1, ZEB2, E-cadherin and miR-200a, miR-200b and miR-141 were analysed by Pearson's correlation. The Receiver Operating Characteristic (ROC) curve analysis was used to evaluate the miRNA expression signature accuracy to discriminate diseased tissues from normal non-diseased tissues. The expression profiles of miR-34c, miR-449a, miR-200a, miR-200b and miR-141 were used as input for ROC analysis by MedCalc software version 12.7.5 (www.medcalc.org). For the joint effect of the best miRNA signature, the multivariate logistic regression model was utilized as described earlier [27]. ROC curve was presented as true positive rate (Sensitivity) plotted in function of the false positive rate (100-Specificity) for different cut-off points. The area under the ROC curve (AUC), describing the ability to distinguish between diseased and non-diseased tissues, was also calculated.

To find a Clinical Score Formula (CSF) that classifies samples into healthy and endometriosis group based on the expression levels of miR-449a, miR-200a and miR-200b, linear soft margin Support Vector Machines classifier was trained using package e1071 in statistics language and environment R (version 3.0.1). Regularization parameter C was chosen by performing a grid search to find the value with lowest 10-fold cross-validation error.

## Results

### High-Throughput miRNA sequencing

miRNA fraction of five peritoneal endometriotic lesions, four matched adjacent healthy tissues (two lesions originating from the same ligament were matched with one respective adjacent healthy tissue) and two eutopic endometrial samples were sequenced using Illumina high throughput sequencing technology. On average, 2.2 million raw reads were obtained from each studied sample, of which 2.1 million (94%) had high quality scores (≥20) according to the FastQC program. Approximately 68% of the good quality score sequencing reads mapped to the human genome and 87% of them aligned to the mature human miRNA sequences in miRBase release 18. Averagely 354 (ranging from 291 to 417) mature miRNAs above 5 read counts and 596 (ranging from 466 to 759) with at least 1 read count per sample mapped to miRBase 18.

The list of expressed miRNAs was highly similar in two eutopic endometria of endometriosis patients. Two most abundant miRNAs in endometrial samples, miR-10b-5p and miR-143-3p, constituted 25.0% and 24.2% of total reads in samples E47 and E101 (Table S5), respectively. Although the lists of the most abundantly expressed miRNAs were comparable, the read counts of the same miRNAs in different endometria showed a wide range of variation probably indicating the inter-individual variability together with the influence of the menstrual cycle phase (proliferative vs. secretory phase endometrium).

**Figure 1:**
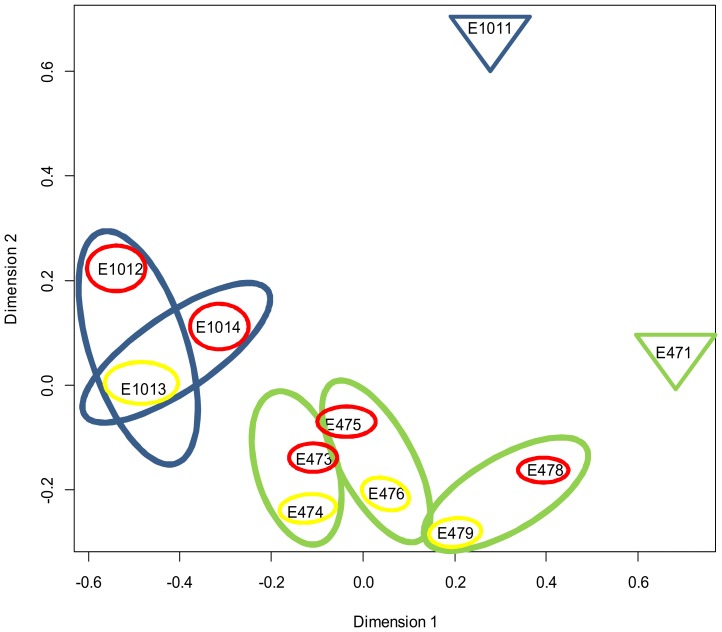
Clustering analysis of the miRNA counts in eutopic and ectopic tissue samples. The edgeR plotMDS function was used to produce multi-dimensional scaling (MDS) plot in which distances reflect biological coefficient of variation between the studied samples. Dimension 1 represents different tissue types (endometria, healthy tissues, endometriotic lesions) while dimension 2 denotes different patients. Blue and green colours indicate different patients. Red circles represent endometriotic lesions, yellow circles adjacent healthy tissues and triangles with patient-specific colour denote respective endometria. The tissue samples presented in figure are following: patient E101 (blue)- endometrium E1011, lesions E1012, E1014, matched healthy tissue E1013; patient E47 (green) - endometrium E471, lesions E473, E475, E478, matched healthy tissues E474, E476, E479.

miRNA expression pattern in endometriotic lesions and adjacent healthy tissues showed a remarkable overlap between matching tissue pairs, and all lesions and adjacent healthy tissues of the same patient revealed high similarity. The MDS plot (Figure 1) demonstrated that all studied tissue pairs of the same patient clustered closer to each other than to the respective endometrium. The list of 15 most abundantly expressed miRNAs in lesions and adjacent healthy tissues is presented in Table S6. Four most abundant miRNAs, expressed both in endometriotic lesions and adjacent healthy tissues, miR-143-3p, miR-22-3p, miR-99a-5p and miR-100-5p, constituted on average 60% of the total reads.

A total of eight potential new miRNA candidates from eutopic/ectopic endometrium and healthy tissues were identified by miRDeep2 (complete list of novel miRNAs is provided in Table S7). Only two out of eight potential miRNAs (miRNA IDs: chr19_39857, chr19_34307) were present either in both endometria and/or in most of the lesions/healthy tissue samples. However, none of the potential novel miRNAs showed specific expression characteristic only to endometriotic lesions.

The edgeR bioinformatics tool was used to compare miRNA read counts between the samples. The comparison of lesions to healthy tissues identified two miRNAs: miR-34c (*P*<0.0001; FDR adjusted *P*-value 0.005, fold change in log2 scale = 4.09) and miR-449a (*P*<0.0001; FDR adjusted *P*-value 0.005, fold change in log2 scale = 4.95) that were significantly up-regulated in endometriotic lesions compared to healthy tissues. The counts of both miRNAs in lesions and healthy tissues were remarkably lower than in eutopic endometria (Table S8).

### qRT-PCR analysis of miR-34c, miR-449a, miR-200a, miR-200b and miR-141

**Figure 2:**
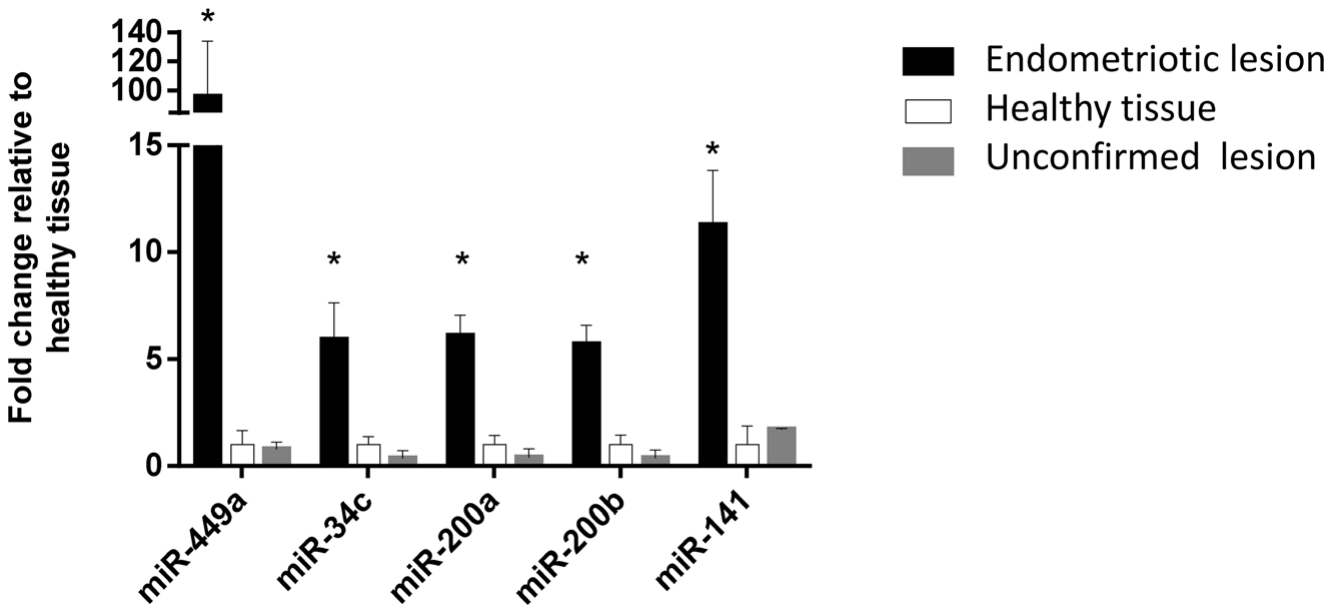
The expression of selected miRNAs in endometriotic lesions (n = 22), histologically unconfirmed lesions (n = 10) and healthy tissues (n = 14) using qRT-PCR. The fold changes for endometriotic lesions and unconfirmed lesions are calculated relative to healthy tissues using the 2^−ΔΔCT^ method [26]. The error bars denote SEM (standard error of the mean). * Denotes comparison of endometriotic lesions with healthy tissues, *P*<0.0001.

Two miRNAs, miR-449a and miR-34c, showing a significantly different expression profile between peritoneal endometriotic lesions and healthy tissues according to the sequencing data were selected for experimental validation. Furthermore, sequencing data of patient E47 tissues showed a trend to higher expression of miR-200 family members miR-200a, miR-200b and miR-141 in lesions compared to healthy tissues (Table S8). Based on these results and the data from the literature [4], [5], [14] suggesting differential expression of these three miRNAs in endometriosis, miR-200a, miR-200b, and miR-141 were additionally selected for validation analysis. The expression of these five miRNAs was validated in 22 diseased and 24 non-diseased tissue samples using TaqMan qRT-PCR. Non-diseased tissue samples were divided into two groups – adjacent healthy tissues (n = 14) and seemingly endometriotic lesions that upon subsequent histological examination showed no evidence of endometriosis (n = 10). Although the sequencing analysis showed low counts for these miRNAs in lesions and healthy tissues, the validation analysis by qRT-PCR revealed well-detectable expression levels. Average Ct values for miR-34c, miR-449a, miR-200a, miR-200b, miR-141, and endogenous controls RNU44 and RNU48 in endometriotic lesions were 25.0, 24.4, 23.2, 24.5, 22.9, 23.8, 19.4, respectively and in non-diseased tissues 29.4, 29.6, 27.0, 28.4, 27.3, 24.7, 20.4, respectively. The respective average ΔCt values (Ct = miRNA Ct value minus average Ct value of RNU44 and RNU48) for miR-449a, miR-34c, miR-200a, miR-200b and miR-141 were 7.4, 5.5, 3.8, 5.0, 4.2 for healthy tissues, 8.5, 7.4, 5.5, 6.8, 5.4 for histologically unconfirmed lesions and 2.3, 3.9, 1.5, 2.9, 1.3 for endometriotic lesions. The results of qRT-PCR indicated that adjacent healthy tissues and histologically unconfirmed lesion-like tissue samples showed very similar expression levels of the tested miRNAs (all *P*>0.05, Figure 2). However, all studied miRNAs showed significantly higher expression in endometriotic lesions (FC for miR-449a = 95.7, miR-34c = 5.9, miR-200a = 6.0, miR-200b = 5.7 and miR-141 = 11.2) compared to healthy tissue samples (all *P*<0.0001). When expression data of healthy tissue samples and unconfirmed lesions–like tissues were combined as one group, even more significant differences were seen between lesions and the non-diseased tissue group (FC for miR-449a = 129.9, miR-34c = 10.2, miR-200a = 10.5, miR-200b = 10.2 and miR-141 = 16.8; *P*<0.0001). As the endometrial miRNA expression profile, at least in part, is shown to be influenced by the menstrual cycle [28], [29], [30] the miRNA expression levels in lesions and non-diseased tissues were also analysed taking into account the menstrual cycle phase. Data analysis showed that miRNA expression in studied tissues was not influenced by the menstrual cycle phase (proliferative vs. secretory) (*P*>0.05).

#### miR-34c, miR-449a, miR-200a, miR-200b and miR-141 expression in the endometrium of endometriosis patients and healthy women

To test whether the expression of miR-34c, miR-449a, miR-200a, miR-200b and miR-141 is dysregulated in the endometrium of endometriosis patients, the endometria of nine patients with endometriosis and eight endometria of healthy women were analysed by qRT-PCR. The results revealed no significant expression level differences between the tested endometria (all *P*>0.05).

#### miR-34c, miR-449a, miR-200a, miR-200b and miR-141 expression in FACS sorted epithelial and stromal cells

To determine whether the expression of these miRNAs was higher in stromal or epithelial cells, FACS sorted pure fractions of endometrial epithelial and stromal cells originating from five endometria from healthy women were used. Data analysis revealed significantly higher expression of studied miRNAs in epithelial cells compared to stromal cells (FC for miR-449a = 86.1, miR-34c = 16.7, miR-200a = 52.4, miR-200b = 44.6 and miR-141 = 68.4; *P*<0.0001), (Figure S1).

### E-cadherin, ZEB1 and ZEB2 expression levels in endometriotic lesions and adjacent healthy tissues

The miR-200 family has been shown to target a complex network of important transcription regulators like ZEB1 and ZEB2 which are transcriptional repressors for E-cadherin [31]. The members of this complex regulate two essentially important biological processes – cell migration and EMT, which are supposed to be crucial events for the development of endometriosis [15]. To determine the differences of E-cadherin, ZEB1 and ZEB2 expression between endometriotic lesions and adjacent healthy tissues, the mRNA levels were studied in 12 paired diseased and non-diseased tissue samples. E-cadherin showed statistically higher expression in endometriotic lesions compared to healthy tissues (t-test, *P* = 0.01) but there were no significant differences in ZEB1 and ZEB2 expression levels between the diseased and non-diseased samples (t-test, *P*>0.05). To detect any correlations between miR-200a, miR-200b and miR-141, and ZEB1, ZEB2, and E-cadherin expression levels, the diseased and non-diseased tissue samples were analysed as one group. The results showed a significant negative correlation between ZEB2 and miR-200a (r = −0.43, *P* = 0.03), miR-200b (r = −0.49, *P* = 0.01), and miR-141 (r = −0.45, *P* = 0.02) expression, and a positive correlation between E-cadherin and miR-200a (r = 0.77, *P*<0.001), miR-200b (r = 0.69, *P*<0.001), and miR-141 (r = 0.57, *P* = 0.02) expression. There was no significant correlation between miR-200 family expression and ZEB1 mRNA levels.

### Diagnostic potential of miR-34c, miR-449a, miR-200a, miR-200b and miR-141

**Figure 3:**
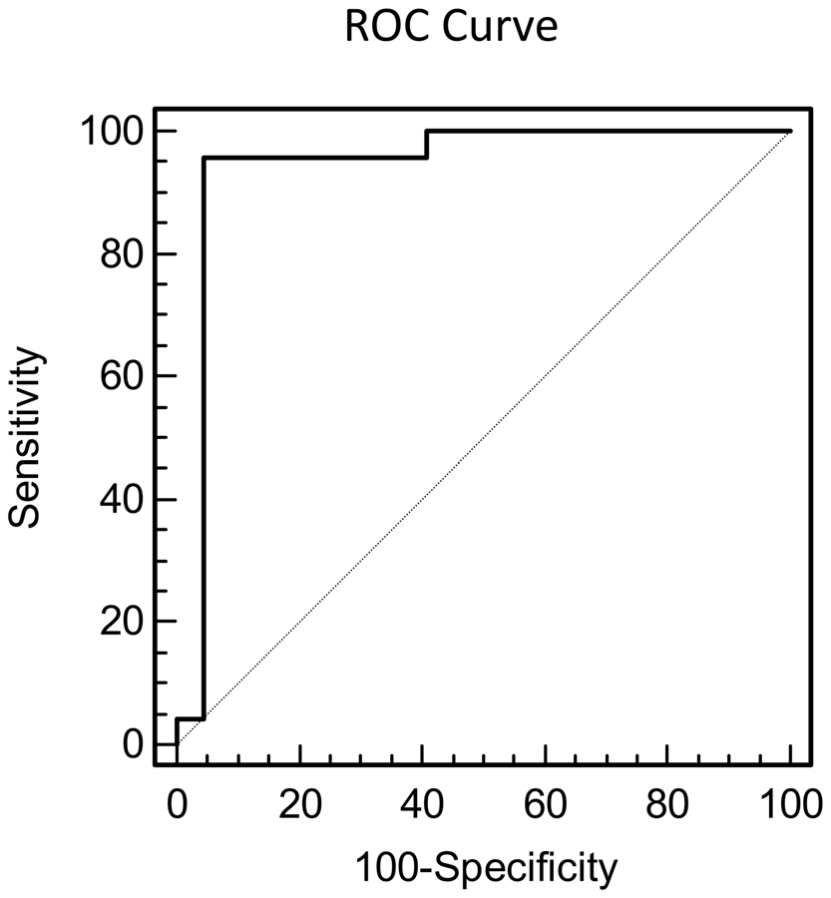
Receiver operating characteristic (ROC) curve analysis for combined signature of miR449a/200a/200b. The miRNA expression signature of miR-449a/200a/200b discriminated endometriotic lesions (n = 22) from non-diseased tissues (n = 24) with 95.8% sensitivity and 95.4% specificity. Area under the ROC curve (AUC) is 0.95 (95% CI, 0.83–0.99).

As the level of investigated miRNAs was much higher in endometriotic lesions than healthy tissues we tested their ability to discriminate between endometriotic lesions (n = 22) and non-diseased tissues (n = 24). The ROC analysis revealed that all miRNAs enabled a correct classification of diseased and non-diseased tissues (AUC values for miR-449a, miR-34c, miR-200a, miR-200b and miR-141 were 0.92, 0.81, 0.91, 0.90, and 0.83, respectively). The combined signature of five miRNAs showed high sensitivity (91.7%) and specificity (95.5%), (AUC = 0.94, CI 0.83–0.99) but the subset of three miRNAs – miR-449a/miR-200a/miR-200b – showed the best discriminating power with the highest sensitivity (95.8%) and specificity (95.5%) (AUC = 0.95, CI 0.83–0.99) (Figure 3).

Based on the expression levels of miR-449a, miR-200a and miR-200b the Clinical Score Formula (CSF) score was calculated, classifying the tissue biopsies into diseased and non-diseased groups.
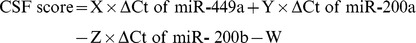



In CSF score X, Y, Z and W denote the coefficients determined by the classifier algorithm which are based on the already analysed tissue samples, while ΔCt is a threshold value difference of target and reference miRNAs of the study samples. More specifically, X, Y and Z (parameters for each miRNA) and W (intercept) are found by training a Support Vector Machine (SVM) classifier which finds a linear combination of the miRNAs that best separates endometriotic lesions from healthy tissues. According to the CSF score, values lower than 0 indicate that the studied tissue samples are endometriotic lesions, and a score above 0 indicates that the studied tissue samples are disease-free. The CSF score enabled to distinguish endometriotic tissue samples from lesion-like samples with very high classification accuracy (>95). The advantage of using CSF scoring is that once the “non-diseased” tissue samples' miRNA levels have been established, these levels can be used as a basis for comparison without the need to rerun the non-diseased samples.

## Discussion

In the current study, high throughput sequencing was used to investigate the miRNome of peritoneal endometriotic lesions. To the best of our knowledge, this is the first miRNA transcriptome study employing a novel strategy including peritoneal endometriotic lesions, macroscopically healthy peritoneal tissue adjacent to lesions and endometria of the same patients to determine the miRNA expression profile characteristic to lesions. We established a signature of five miRNAs that reflects the presence of endometrial cells in peritoneal endometriotic lesion biopsies.

To date, many endometriosis studies have focused on the detection of potential disease-associated miRNAs by examining eutopic and ectopic endometria [4], [5], [6], [7], [8], [9], [10], [11], [12], [13], [14]. Recent review by Gilabert-Estelles et al. has summarized the findings of the previous studies and found only a subset of miRNAs that have been found relevant in pathogenesis of endometriosis in more than two studies [3]. The discordances between the results of these studies could reflect different study strategies as some of the studies have compared the miRNA profile of ectopic lesions to eutopic endometria of endometriosis patients or disease-free women while the others have studied only endometria of patients and controls. However, endometriotic lesion biopsies usually do not contain only endometriotic cells but rather are the mixture of endometriotic cells and surrounding tissue in various proportions. There is clear evidence that different tissue types have their unique miRNA profiles [32], therefore the comparisons between eu- and ectopic endometria biopsy samples would not reveal authentic lesion-specific miRNA expression profile. Based on this assumption and our MDS plot data, in this study we compared the peritoneal endometriotic lesions to adjacent healthy tissues to diminish the impact of surrounding tissue on miRNA expression and excluded endometria from further analysis. Our data analysis revealed only a few significantly differently expressed miRNAs in lesions and thus we believe that surrounding peritoneal tissue miRNA expression masks most of the miRNA expression differences that could originate from endometriotic cells. We propose that the expression levels of miRNAs in endometrial cells and surrounding tissue need to be remarkably different in order to reveal any distinct miRNAs originating from a small proportion of endometrial cells inside the lesion.

Using the deep sequencing approach, we detected two miRNAs, miR-34c and miR-449a, which were significantly up-regulated in endometriotic lesion biopsies compared to healthy peritoneal tissues. Our validation study showed an almost six-fold higher expression of miR-34c, and a 95-fold higher expression of miR-449a in lesions compared to non-diseased tissues. miR-34c and miR-449a are classified as one family because of their highly similar secondary structures and seed sequence [33]. It has been suggested that members of this family are capable of mediating cell cycle arrest and apoptosis via p53 and thereby could contribute to pathologic processes (reviewed in [33]). To date, there is no data about the relevance of miR-449a in endometriosis but many cancer studies have shown down-regulation of miR-449a in cancer cell lines and solid tumours (reviewed [34]). Some earlier studies have shown altered expression of miR-34c in ectopic endometrium compared to eutopic or control endometrium [5], [14] suggesting miR-34c involvement in endometriosis development. These studies used ectopic endometrial samples from different anatomical locations (peritoneal lesions and ovarian endometriomas); however, significant down-regulation of miR-34c in ectopic lesions was revealed in both cases. The sequencing data obtained in current study are in good concordance with previous findings showing higher miR-34c counts in eutopic endometria compared to endometriotic lesions. However, none of the previous studies have compared the miRNA profile of lesion biopsies to the healthy peritoneal tissues and we suggest that the differences between miR-34c and miR-449a abundance in lesions and eutopic endometria reflect the presence of a limited number of endometrial cells in peritoneal tissue, rather than expression differences between endometrial cells in different locations.

Interestingly, down-regulation of miR-34c expression was also shown when eutopic endometria of endometriosis patients were compared to control endometria [9]. We also compared the expression levels of miR-34c in eutopic endometria of endometriosis patients to endometria of healthy women but contrary to the findings reported by Burney et al. [9] the results of this study showed no significant differences. The possible reason for this discrepancy may be different patient selection criteria, as Burney et al. investigated women with moderate-severe endometriosis and our patient group included also patients with minimal-mild endometriosis. Furthermore, the used control groups were different (patients with leiomyomas vs. healthy women in our study).

The aberrant expression of miR-200 family miRNAs has been demonstrated in peritoneal lesions [5] as well as in endometriomas [4], [14]. All three previous studies [4], [5], [14] showed significantly reduced expression levels of miR-200a and miR-200b in ectopic lesions compared to eutopic and control endometria, and Ohlsson Teague et al. [5] also demonstrated lowered miR-141 levels in peritoneal lesions. Ohlsson Teague et al. [35] have suggested the involvement of miR-200 family members in endometriosis pathogenesis as the low miR-200 expression and enhanced TGFβ activity may promote EMT and a migratory mesenchymal phenotype in endometriotic tissues. During the EMT process, cells lose their epithelial features and obtain mesenchymal characteristics, and this process is regulated by the double-negative regulatory ZEB/miR-200 feedback loop [36]. The miR-200 family members are cooperatively involved in down-regulation of ZEB1 and ZEB2 and up-regulation of E-cadherin expression levels, and induction of mesenchymal phenotype characteristic to EMT is dependent upon repression of the miR-200 family [37]. miR-200 family together with E-cadherin are shown to be good indicators of the epithelial nature of cells, and there is strong evidence that overexpression of ZEB1/ZEB2 and the subsequent loss of E-cadherin expression initiate pathological processes [31], [38], [39]. Thereby, based on the data from the literature [4], [5], [14] and also supported by our sequencing results, three additional miRNAs (miR-200a, miR-200b and miR-141) were investigated and confirmed to be expressed at significantly higher levels in lesions compared to healthy tissues. However, when the expression levels of miR-200a, miR-200b and miR-141 were measured in eutopic endometria of patients with endometriosis and healthy women, no differences were seen. Like in the case of miR-34c and miR-449a, the sequencing read counts of miR-200a, miR-200b and miR-141 were higher in eutopic endometria than in endometriotic lesions and we propose that similar to miR-34c and miR-449a, the expression signature of these three miRNAs is not related to different characteristics of endometrial cells in eu- or ectopic locations, but rather reflects the presence of endometrial cells in peritoneal tissue. It is well-proven that the endometriotic epithelial cells in lesions originate from endometrial epithelial cells [15]. Our data showed that all five investigated miRNAs were more highly expressed in FACS sorted endometrial epithelial than in stromal cells and therefore we propose that these miRNAs foremost reflect the presence of epithelial cells in endometriotic lesions.

As the results of our validation study showed significantly higher expression of miR-200a, miR-200b and miR-141 in endometriotic lesions compared to healthy tissues, we also determined the expression of miR-200 family targets ZEB1, ZEB2 and E-cadherin in lesions and healthy peritoneum. We observed significantly higher E-cadherin levels but there were no differences in ZEB1 and ZEB2 expression in lesions compared to healthy tissues. However, we found a significant negative correlation between the expression levels of ZEB2 mRNA and investigated miR-200 family members that are in good concordance with the previous study [31]. The possible reason why we saw E-cadherin up-regulation could be the extent of epithelial endometrial cells in lesions, as E-cadherin is shown to be expressed mainly in epithelial cells [40] and ZEB1 and/or ZEB2 are expressed primarily in stromal cells [41], [42]. Park et al., (2008) studied 59 cell lines and detected that endogenous miR-200 targeted the ZEB2 mRNA so that not a single cell line expressed both miR-200 and ZEB2. Therefore, the higher expression of ZEB1 and ZEB2 could be detected only in cells where the expression of miR-200 is low, as in endometrial stromal cells, and it is very likely that if only a limited number of stromal cells are present in endometriotic lesion, the difference between ZEB1/ZEB2 expression in lesions and healthy tissues is undetectable.

It has been shown that almost half of the surgical specimens removed during laparoscopy are not confirmed in the following histological assessments [43], [44], [45]. The usual histological findings of lesion-like structures include fibrosis, inflammatory changes and normal peritoneum [46]. Therefore, in the light of our results showing significantly higher levels of some miRNAs in biopsied lesions, we decided to test the applicability of these markers to discriminate between true endometriotic lesions and lesion-like tissue structures. The expression signature of three miRNAs, miR-449a/miR-200a/miR-200b, showed the best discrimination power to separate the endometriotic lesions from the non-diseased tissues with a sensitivity of 95.8% and specificity of 95.5%. Based on this data, the CSF model was developed, enabling evaluation of the biopsied tissue samples without the need for repeated use of non-diseased tissues. These three miRNAs have a great potential to be used as new molecular diagnostic markers instead of histological assessment to confirm the presence of endometrial cells in biopsied samples. During the routine histological assessment, several tissue sections from different parts of the biopsy are examined, whereas a molecular miRNA-based test would allow the evaluation of the entire biopsy at once, giving accurate diagnosis with high sensitivity and specificity. However, although the miRNA-based test is promising, some of the samples (one sample out of 24 non-diseased tissues and one out of 22 diseased tissues) clustered to the wrong group. The clustering of the healthy sample to the endometriosis group may be caused by missed endometriosis during histological examination or by unnoticed small lesions on visually healthy peritoneum. The presence of endometrial tissue structures in visually healthy peritoneal tissues has been reported previously [47], [48]. Also, a recent study by Khan et al. [49] confirmed the presence of endometrial stromal and/or epithelial cells in 15% of the visually healthy peritoneal biopsies originating from endometriosis patients. The false-negative finding, an endometriotic lesion that clustered to the healthy tissue group could be explained by the very small amount of endometrial stromal and epithelial cells inside the lesion. It may also be that all tissue sections containing any endometrial cell structures were used during histological evaluation and the remaining tissue sample that was used for miRNA extraction contained no endometrial cells.

Some limitations of the study should also be mentioned. The number of sequenced samples was moderate and contained samples only from women with severe endometriosis. Therefore, our study was probably underpowered to discover miRNAs with subtle expression differences between the tissues. However, the validation set of 46 diseased and non-diseased samples originating both from women with minimal-mild and moderate-severe endometriosis confirmed the main sequencing results. Additionally, although the FACS experiment showed significantly higher expression of these miRNAs in endometrial epithelial cells, these results cannot be extrapolated to endometriotic lesions without caution as the pelvic environment may induce changes in miRNA expression levels. Future experiments using pure populations of epithelial and stromal cells originating from endometriotic lesions are needed to clarify this issue. Also, it should be pointed out that findings of this study are valid only for peritoneal endometriotic lesions.

In conclusion, we found five miRNAs with elevated expression in peritoneal endometriotic lesions compared to healthy surrounding tissues, enabling also reliable discrimination between diseased and healthy tissues. Furthermore, the results of our study indicated that if examining the entire biopsied sample of endometriotic lesion, the miRNA expression of surrounding tissue masks most of the miRNA profile that originates from the endometriotic tissue. To overcome this issue, future studies evaluating the miRNome of endometriotic lesions should focus on pure cell populations (cell culture studies, stroma and glandular epithelial cells obtained by laser capture microdissection or FACS sorting) instead of studying the entire endometriotic lesion biopsies.

## Supporting Information

Figure S1 **miR-34c, miR-449a, miR-200a, miR-200b and miR-141 expression in FACS sorted epithelial and stromal cells obtained from five healthy women endometria.** Data are represented as the mean ΔCt ± SD (Ct value of miRNA minus average Ct value of RNU44 and RNU48) for each miRNA. The average Ct for investigated miRNAs in stromal cells were higher (Ct values for miR-34c, miR-449, miR-200a, miR-200b and miR-141 were 34.6, 32.7, 31.7, 31.7, 32.8 and 31.9, respectively) than in epithelial cells (respective Ct values were 31.4, 27.1, 26.9, 28.2, 26.7). As the average Ct values of RNUs were lower than of miRNAs, the higher ΔCt values mean lower miRNA expression. Student's t-test showed statistically significant differences between all miRNA expression levels in epithelial and stromal cells (all *P*<0.01).
(TIF)

Table S1 **Clinical characteristics of patients and tissue samples used in the sequencing study.**
(DOCX)

Table S2 **Clinical characteristics of patients and tissue samples used in the miRNA expression validation study.**
(DOCX)

Table S3 **Clinical characteristics of patients and controls used in the endometrium study.**
(DOCX)

Table S4 **Clinical characteristics of healthy women used for endometrial stromal and epithelial cells FACS sorting study.**
(DOCX)

Table S5 **List of most abundant miRNAs in two different endometria.**
(DOCX)

Table S6 **List of 15 most abundant miRNAs expressed in different endometriotic lesions and healthy tissues.**
(DOCX)

Table S7 **The list of potential novel miRNAs proposed by miRDeep2.**
(DOCX)

Table S8 **Read counts of differentially expressed miRNAs in studied samples.**
(DOCX)
