# Supplementary material for: High-Throughput Sequencing Approach Uncovers the miRNome of Peritoneal Endometriotic Lesions and Adjacent Healthy Tissues
Source: PLoS One. 2014 Nov 11;9(11):e112630. doi: 10.1371/journal.pone.0112630 (PMC4227690; doi:10.1371/journal.pone.0112630)
Supplement: Table S1 — Clinical characteristics of patients and tissue samples used in the sequencing study. (DOCX) [file pone.0112630.s002.docx]

| Table S1. Clinical characteristics of patients and tissue samples used in the sequencing study | | | | | | | |
| --- | --- | --- | --- | --- | --- | --- | --- |
| **Patient ID** | **Age** | **BMI** | **Menstrual cycle phase** | **Endometriosis stage** | **Analysed tissues** | | |
| E47 | 27 | 22 | proliferative | III-IV | **Endometrium** | **Endometriotic lesion location** | **Matched healthy tissue** |
|  |  |  |  |  | E47.1/E471* | E47.3/E473* Cavum Douglas | E47.4/E474* Cavum Douglas |
|  |  |  |  |  |  | E47.5/E475* Lig. latum dex. | E47.6/E476* Lig. latum dex. |
|  |  |  |  |  |  | E47.8/E478 *Lig.sacrouterina sin. | E47.9/E479* Lig.sacrouterina sin. |
| E101 | 28 | 22 | secretory | III-IV | E101.1/E1011* | E101.2/E1012* Lig.sacrouterina dex. | E101.3/E1013* Lig.sacrouterinadex. |
|  |  |  |  |  |  | E101.4/E1014* Lig.sacrouterina dex. |  |

*denotes different labelling of the same samples
