# Supplementary material for: High-Throughput Sequencing Approach Uncovers the miRNome of Peritoneal Endometriotic Lesions and Adjacent Healthy Tissues
Source: PLoS One. 2014 Nov 11;9(11):e112630. doi: 10.1371/journal.pone.0112630 (PMC4227690; doi:10.1371/journal.pone.0112630)
Supplement: Table S2 — Clinical characteristics of patients and tissue samples used in the miRNA expression validation study. (DOCX) [file pone.0112630.s003.docx]

| Table S2. Clinical characteristics of patients and tissue samples used in the miRNA expression validation study. | | | | | | | | | |
| --- | --- | --- | --- | --- | --- | --- | --- | --- | --- |
| **Patient ID** | **Age** | **BMI** | **Menstrual cycle phase** | **Endometriosis stage** | **Endometriotic lesion**  **location** | | **Non-diseased tissues (n=24)** | | |
|  |  |  |  |  |  | **Healthy tissue**  **location** | | **Unconfirmed lesion**  **location** | |
| E47 | 27 | 22 | proliferative | III-IV | *E47.3 Cavum Douglas | | *E47.4 Cavum Douglas | - |  |
|  |  |  |  |  | *E47.5 Lig. latum dex | *E47.6 Lig. latum dex. | | - | |
|  |  |  |  |  | *E47.8 Lig.sacrouterina sin. | *E47.9 Lig.sacrouterina sin. | | - | |
| E101 | 28 | 22 | secretory | III-IV | *E101.2 Lig.sacrouterina dex. | *E101.3Lig.sacrouterina dex. | | - | |
|  |  |  |  |  | E101.4 Lig.sacrouterina dex. | E101.5 Lig.sacrouterina sin. | |  |  |
| E2 | 35 | 23 | proliferative | II | E2.3 Lig.sacrouterina dex. | - | | - | |
| E10 | 32 | n/a | proliferative | IV | - | - | | El10.2 Uteral superficial | |
| E11 | 36 | 21 | secretory | III | - | - | | El11.2 Lig.sacrouterina dex. | |
|  |  |  |  |  |  |  |  | El11.3 Fossa ovarica | |
| E15 | 42 | 18 | proliferative | III | E15.2 Lig.sacrouterina sin. | - | | E15.4 Uteral superficial | |
| E16 | 37 | 26 | proliferative | III | E16.4 Lig.sacrouterina sin. | - | | - | |
|  |  |  |  |  | E16.6 Cavum Douglas | - | | - | |
| E17 | 30 | 19 | proliferative | III | - | - | | E17.6 Lig.sacrouterina dex. | |
|  |  |  |  |  |  |  |  | E17.7 Uteral superficial | |
| E18 | 31 | 20 | proliferative | III | E18.2 Lig.sacrouterina sin. | - | | - | |
|  |  |  |  |  | E18.3 Uteral superficial | - | | - | |
| E20 | 41 | 26 | proliferative | III | - | - | | E20.4 Uteral superficial | |
| E40 | 33 | 22 | secretory | III-IV | *E40.2 Lig. sacrouterina | *E40.4 Lig.sacrouterina | | - | |
| E41 | 39 | 25 | proliferative | I-II | *E41.2 Lig.sacrouterina dex. | *E41.4 Lig.sacrouterina dex. | | - | |
| E52 | 30 | 20 | proliferative | II | E52.4 Lig.sacrouterina sin. |  | | - | |
| E81 | 33 | 19 | secretory | I-II | *E81.2 Lig.sacrouterina | *E81.3 Lig.sacrouterina | | - | |
| E100 | 39 | 27 | secretory | I-II | *E100.2 Lig.sacrouterina sin. | *E100.4 Lig.sacrouterina dex. | | - | |
| E104 | 34 | 21 | secretory | I-II | *E104.2 Fossa ovarica dex. | *E104.3 Fossa ovarica dex. | | - | |
|  |  |  |  |  | *E104.4 Lig. sacrouterina sin. | *E104.5 Lig. sacrouterina sin. | | - | |
| E112 | 27 | 23 | secretory | I-II | *E112.2 Lig.sacrouterina sin. | *E112.3 Lig.sacrouterina dex. | | - | |
| E123 | 36 | 21 | proliferative | III | - | - | | E123.2 Lig.sacrouterina sin. | |
| E138 | 29 | 23 | proliferative | III | E138.2 Cavum Douglas | - | |  | |
| E142 | 31 | 20 | proliferative | IV | - | - | | E142.3 Fossa ovarica | |
|  |  |  |  |  |  |  |  | E142.5 Uteral superficial | |
| E146 | 27 | 26 | secretory | - | - | E146.2 Lig.sacrouterina | | - | |
| E147 | 39 | 29 | secretory | I | *E147.3 Lig. sacrouterina sin. | *E147.4 Lig.sacrouterina dex. | | - | |
| E148 | 20 | 24 | secretory | III | E148.2 Lig.sacrouterina | - | | - | |

*Tissue samples used in E-cadherin, ZEB1 and ZEB2 mRNA expression study
