# Supplementary material for: High-Throughput Sequencing Approach Uncovers the miRNome of Peritoneal Endometriotic Lesions and Adjacent Healthy Tissues
Source: PLoS One. 2014 Nov 11;9(11):e112630. doi: 10.1371/journal.pone.0112630 (PMC4227690; doi:10.1371/journal.pone.0112630)
Supplement: Table S3 — Clinical characteristics of patients and controls used in the endometrium study. (DOCX) [file pone.0112630.s004.docx]

| **Patient ID** | **Age** | **BMI** | **Menstrual cycle phase** | **Endometriosis stage** |
| --- | --- | --- | --- | --- |
| E104 | 34 | 17 | secretory | I |
| E106 | 28 | n/a | secretory | IV |
| E121 | 26 | 30 | secretory | III |
| E66 | 34 | 21 | secretory | I |
| E105 | 38 | 22 | secretory | III |
| E107 | 26 | 23 | secretory | II |
| E71 | 32 | 24 | secretory | III |
| E82 | 28 | 23 | secretory | I |
| E119 | 32 | 20 | secretory | III |
| **Healthy controls** | | | | |
| K1 | 30 | 23 | secretory | - |
| K2 | 35 | 22 | secretory | - |
| K3 | 25 | 23 | secretory | - |
| K4 | 34 | 22 | secretory | - |
| K5 | 32 | 21 | secretory | - |
| K6 | 34 | 19 | secretory | - |
| K7 | 31 | 18 | secretory | - |
| K8 | 25 | 20 | secretory | - |

Table S3. Clinical characteristics of patients and controls used in the endometrium study
