# Supplementary material for: High-Throughput Sequencing Approach Uncovers the miRNome of Peritoneal Endometriotic Lesions and Adjacent Healthy Tissues
Source: PLoS One. 2014 Nov 11;9(11):e112630. doi: 10.1371/journal.pone.0112630 (PMC4227690; doi:10.1371/journal.pone.0112630)
Supplement: Table S4 — Clinical characteristics of healthy women used for endometrial stromal and epithelial cells FACS sorting study. (DOCX) [file pone.0112630.s005.docx]

| **Participant ID** | **Age** | **BMI** | **Menstrual cycle phase** |
| --- | --- | --- | --- |
| K9 | 26 | 23 | secretory |
| K10 | 28 | 21 | secretory |
| K11 | 29 | 20 | secretory |
| K12 | 36 | 21 | secretory |
| K13 | 26 | 29 | secretory |

Table S4. Clinical characteristics of healthy women used for endometrial stromal and epithelial cells FACS sorting study.
