# Supplementary material for: High-Throughput Sequencing Approach Uncovers the miRNome of Peritoneal Endometriotic Lesions and Adjacent Healthy Tissues
Source: PLoS One. 2014 Nov 11;9(11):e112630. doi: 10.1371/journal.pone.0112630 (PMC4227690; doi:10.1371/journal.pone.0112630)
Supplement: Table S5 — List of most abundant miRNAs in two different endometria. (DOCX) [file pone.0112630.s006.docx]

| Table S5. List of most abundant miRNAs in two different endometria | | | | | |
| --- | --- | --- | --- | --- | --- |
| **E47.1** | **Number of reads** | **% of total reads** | **E101.1** | **Number of reads** | **% of total reads** |
| **hsa-miR-10b-5p** | 131635 | 14.46 | **hsa-miR-10b-5p** | 87155 | 12.20 |
| **hsa-miR-143-3p** | 95595 | 10.50 | **hsa-miR-143-3p** | 85961 | 12.03 |
| **hsa-miR-21-5p** | 91396 | 10.04 | **hsa-miR-10a-5p** | 48546 | 6.80 |
| **hsa-miR-10a-5p** | 54798 | 6.02 | **hsa-miR-146b-5p** | 41559 | 5.82 |
| **hsa-miR-99a-5p** | 39985 | 4.39 | **hsa-miR-99a-5p** | 39644 | 5.55 |
| hsa-miR-451a | 34394 | 3.78 | **hsa-miR-100-5p** | 39476 | 5.53 |
| **hsa-miR-146b-5p** | 33761 | 3.71 | **hsa-miR-21-5p** | 34419 | 4.82 |
| hsa-miR-26a-5p | 32122 | 3.53 | **hsa-miR-27b-3p** | 29050 | 4.07 |
| **hsa-miR-27b-3p** | 30451 | 3.34 | **hsa-let-7g-5p** | 23733 | 3.32 |
| **hsa-miR-199a-3p** | 22867 | 2.51 | **hsa-let-7i-5p** | 23552 | 3.30 |
| **hsa-miR-199b-3p** | 22867 | 2.51 | hsa-miR-186-5p | 18863 | 2.64 |
| **hsa-miR-24-3p** | 16106 | 1.77 | hsa-miR-320a | 16527 | 2.31 |
| **hsa-miR-100-5p** | 14648 | 1.61 | hsa-miR-21-3p | 16163 | 2.26 |
| hsa-miR-144-3p | 14122 | 1.55 | **hsa-miR-22-3p** | 16074 | 2.25 |
| **hsa-let-7g-5p** | 12759 | 1.40 | **hsa-miR-27a-3p** | 12672 | 1.77 |
| **hsa-miR-27a-3p** | 11901 | 1.31 | **hsa-miR-24-3p** | 11448 | 1.60 |
| **hsa-let-7i-5p** | 10100 | 1.11 | **hsa-miR-199a-3p** | 10935 | 1.53 |
| hsa-miR-199b-5p | 10020 | 1.10 | **hsa-miR-199b-3p** | 10935 | 1.53 |
| **hsa-miR-22-3p** | 9248 | 1.02 | hsa-miR-30a-5p | 9877 | 1.38 |
| hsa-miR-125b-5p | 8967 | 0.98 | hsa-miR-378a-3p | 9403 | 1.32 |
| All miRNAs marked in bold are overlapping between different endometrial samples | | | | | |
