# Supplementary material for: High-Throughput Sequencing Approach Uncovers the miRNome of Peritoneal Endometriotic Lesions and Adjacent Healthy Tissues
Source: PLoS One. 2014 Nov 11;9(11):e112630. doi: 10.1371/journal.pone.0112630 (PMC4227690; doi:10.1371/journal.pone.0112630)
Supplement: Table S6 — List of 15 most abundant miRNAs expressed in different endometriotic lesions and healthy tissues. (DOCX) [file pone.0112630.s007.docx]

| Table S6. List of 15 most abundant miRNAs expressed in different endometriotic lesions and healthy tissues | | | | | | | | | | | | |  |
| --- | --- | --- | --- | --- | --- | --- | --- | --- | --- | --- | --- | --- | --- |
|  |  | | | |  |  | |  | |  |  | |  |
| **Endometriotic lesions** | | | | |  |  | |  | |  |  | |  |
| **Patient ID/number of the sample E47 /47.3** | | | | | | **Patient ID/number of the sample E47 /47.5** | | | | | **Patient ID/number of the sample E47 /47.8** | | |
| **miRNA ID** | | | **Number of reads** | **% of total reads** | | **miRNA ID** | **Number of reads** | | **% of total reads** | | **miRNA ID** | **Number of reads** | **% of total reads** |
| hsa-miR-143-3p | | | 375368 | 41.26 | | hsa-miR-143-3p | 194002 | | 23.01 | | hsa-miR-143-3p | 361980 | 41.18 |
| hsa-miR-22-3p | | | 109178 | 12.00 | | hsa-miR-22-3p | 143116 | | 16.98 | | hsa-miR-21-5p | 108999 | 12.40 |
| hsa-miR-99a-5p | | | 97277 | 10.69 | | hsa-miR-99a-5p | 97783 | | 11.60 | | hsa-miR-10b-5p | 61259 | 6.97 |
| hsa-miR-100-5p | | | 42632 | 4.69 | | hsa-miR-100-5p | 39724 | | 4.71 | | hsa-miR-10a-5p | 32906 | 3.74 |
| hsa-miR-145-5p | | | 35385 | 3.89 | | hsa-miR-10b-5p | 28038 | | 3.33 | | hsa-miR-99a-5p | 22086 | 2.51 |
| hsa-miR-10b-5p | | | 24958 | 2.74 | | hsa-miR-26a-5p | 23049 | | 2.73 | | hsa-miR-100-5p | 20801 | 2.37 |
| hsa-miR-26a-5p | | | 15619 | 1.72 | | hsa-miR-146b-5p | 19511 | | 2.31 | | hsa-miR-26a-5p | 19655 | 2.24 |
| hsa-let-7b-5p | | | 8994 | 0.99 | | hsa-let-7b-5p | 14669 | | 1.74 | | hsa-miR-451a | 18123 | 2.06 |
| hsa-let-7i-5p | | | 8195 | 0.90 | | hsa-let-7i-5p | 14318 | | 1.70 | | hsa-miR-199a-3p | 13821 | 1.57 |
| hsa-let-7a-5p | | | 7689 | 0.85 | | hsa-miR-199a-3p | 13644 | | 1.62 | | hsa-miR-199b-3p | 13821 | 1.57 |
| hsa-miR-125b-5p | | | 7679 | 0.84 | | hsa-miR-199b-3p | 13644 | | 1.62 | | hsa-miR-144-3p | 11883 | 1.35 |
| hsa-let-7g-5p | | | 7525 | 0.83 | | hsa-miR-10a-5p | 12836 | | 1.52 | | hsa-miR-22-3p | 11737 | 1.34 |
| hsa-miR-99b-5p | | | 7463 | 0.82 | | hsa-miR-145-5p | 12482 | | 1.48 | | hsa-miR-27b-3p | 8620 | 0.98 |
| hsa-miR-199a-3p | | | 7385 | 0.81 | | hsa-let-7a-5p | 10474 | | 1.24 | | hsa-let-7a-5p | 8543 | 0.97 |
| hsa-miR-199b-3p | | | 7385 | 0.81 | | hsa-let-7g-5p | 10225 | | 1.21 | | hsa-miR-146b-5p | 7568 | 0.86 |
|  | | |  |  | |  |  | |  | |  |  |  |
| **Patient ID/number of the sample E101 /101.2** | | | | | | **Patient ID/number of the sample E101 /101.4** | | | | |  |  |  |
| **miRNA ID** | | | **Number of reads** | | **% of total reads** | **miRNA ID** | | **Number of reads** | | **% of total reads** |  |  |  |
| hsa-miR-22-3p | | | 279494 | | 38.39 | hsa-miR-143-3p | | 388227 | | 41.93 |  |  |  |
| hsa-miR-143-3p | | | 188306 | | 25.86 | hsa-miR-99a-5p | | 134583 | | 14.54 |  |  |  |
| hsa-miR-320a | | | 31503 | | 4.33 | hsa-miR-22-3p | | 109926 | | 11.87 |  |  |  |
| hsa-miR-99a-5p | | | 22190 | | 3.05 | hsa-miR-100-5p | | 106087 | | 11.46 |  |  |  |
| hsa-miR-145-5p | | | 14716 | | 2.02 | hsa-miR-145-5p | | 12535 | | 1.35 |  |  |  |
| hsa-miR-378a-3p | | | 14612 | | 2.01 | hsa-miR-143-5p | | 9743 | | 1.05 |  |  |  |
| hsa-miR-143-5p | | | 13449 | | 1.85 | hsa-miR-320a | | 8957 | | 0.97 |  |  |  |
| hsa-miR-127-3p | | | 12837 | | 1.76 | hsa-miR-127-3p | | 7510 | | 0.81 |  |  |  |
| hsa-miR-100-5p | | | 10814 | | 1.49 | hsa-let-7i-5p | | 7391 | | 0.8 |  |  |  |
| hsa-let-7b-5p | | | 8208 | | 1.13 | hsa-let-7b-5p | | 7312 | | 0.79 |  |  |  |
| hsa-miR-24-3p | | | 6649 | | 0.91 | hsa-miR-26a-5p | | 6775 | | 0.73 |  |  |  |
| hsa-miR-199a-5p | | | 6020 | | 0.83 | hsa-miR-378a-3p | | 6504 | | 0.7 |  |  |  |
| hsa-miR-199a-3p | | | 5876 | | 0.81 | hsa-let-7g-5p | | 6110 | | 0.66 |  |  |  |
| hsa-miR-199b-3p | | | 5876 | | 0.81 | hsa-let-7a-5p | | 6062 | | 0.65 |  |  |  |
| hsa-miR-27b-3p | | | 5345 | | 0.73 | hsa-miR-199a-3p | | 5967 | | 0.64 |  |  |  |
|  | | |  | |  |  | |  | |  |  |  |  |
| **Healthy adjacent tissues** | | | | |  |  | |  | |  |  |  |  |
| **Patient ID/number of the sample E47 /47.4** | | | | | | **Patient ID/number of the sample E47 /47.6** | | | | |  |  |  |
| **miRNA ID** | | **Number of reads** | | | **% of total reads** | **miRNA ID** | | **Number of reads** | | **% of total reads** |  |  |  |
| hsa-miR-143-3p | | 493896 | | | 52.49 | hsa-miR-143-3p | | 123058 | | 13.16 |  |  |  |
| hsa-miR-22-3p | | 116403 | | | 12.37 | hsa-miR-10b-5p | | 99032 | | 10.59 |  |  |  |
| hsa-miR-99a-5p | | 44736 | | | 4.75 | hsa-miR-99a-5p | | 81034 | | 8.67 |  |  |  |
| hsa-miR-145-5p | | 36421 | | | 3.87 | hsa-miR-22-3p | | 59195 | | 6.33 |  |  |  |
| hsa-miR-100-5p | | 14423 | | | 1.53 | hsa-miR-146b-5p | | 53263 | | 5.70 |  |  |  |
| hsa-miR-26a-5p | | 13238 | | | 1.41 | hsa-miR-26a-5p | | 48892 | | 5.23 |  |  |  |
| hsa-let-7a-5p | | 12831 | | | 1.36 | hsa-miR-125b-5p | | 39704 | | 4.25 |  |  |  |
| hsa-miR-10b-5p | | 11664 | | | 1.24 | hsa-miR-21-5p | | 27582 | | 2.95 |  |  |  |
| hsa-let-7b-5p | | 10319 | | | 1.10 | hsa-miR-10a-5p | | 27122 | | 2.90 |  |  |  |
| hsa-miR-21-5p | | 9606 | | | 1.02 | hsa-miR-100-5p | | 25188 | | 2.69 |  |  |  |
| hsa-miR-199a-3p | | 9275 | | | 0.99 | hsa-miR-199a-3p | | 20781 | | 2.22 |  |  |  |
| hsa-miR-199b-3p | | 9275 | | | 0.99 | hsa-miR-199b-3p | | 20781 | | 2.22 |  |  |  |
| hsa-miR-27b-3p | | 8706 | | | 0.93 | hsa-miR-145-5p | | 19978 | | 2.14 |  |  |  |
| hsa-let-7f-5p | | 8161 | | | 0.87 | hsa-miR-191-5p | | 16934 | | 1.81 |  |  |  |
| hsa-miR-24-3p | | 7589 | | | 0.81 | hsa-miR-186-5p | | 16784 | | 1.80 |  |  |  |
|  | |  | | |  |  | |  | |  |  |  |  |
| **Patient ID/number of the sample E47 /47.9** | | | | | | **Patient ID/number of the sample E101 /101.3** | | | | |  |  |  |
| **miRNA ID** | | **Number of reads** | | | **% of total reads** | **miRNA ID** | | **Number of reads** | | **% of total reads** |  |  |  |
| hsa-miR-143-3p | | 539743 | | | 56.32 | hsa-miR-143-3p | | 274187 | | 29.66 |  |  |  |
| hsa-miR-10b-5p | | 97726 | | | 10.20 | hsa-miR-22-3p | | 234385 | | 25.35 |  |  |  |
| hsa-miR-99a-5p | | 40716 | | | 4.25 | hsa-miR-99a-5p | | 107862 | | 11.67 |  |  |  |
| hsa-miR-22-3p | | 30090 | | | 3.14 | hsa-miR-100-5p | | 51870 | | 5.61 |  |  |  |
| hsa-miR-145-5p | | 25268 | | | 2.64 | hsa-miR-145-5p | | 47118 | | 5.10 |  |  |  |
| hsa-miR-100-5p | | 17229 | | | 1.80 | hsa-miR-320a | | 27190 | | 2.94 |  |  |  |
| hsa-miR-21-5p | | 16023 | | | 1.67 | hsa-miR-127-3p | | 17973 | | 1.94 |  |  |  |
| hsa-miR-26a-5p | | 15141 | | | 1.58 | hsa-miR-143-5p | | 17272 | | 1.87 |  |  |  |
| hsa-miR-27b-3p | | 8054 | | | 0.84 | hsa-miR-378a-3p | | 15877 | | 1.72 |  |  |  |
| hsa-miR-24-3p | | 7683 | | | 0.80 | hsa-miR-99b-5p | | 13179 | | 1.43 |  |  |  |
| hsa-miR-10a-5p | | 7289 | | | 0.76 | hsa-let-7b-5p | | 8560 | | 0.93 |  |  |  |
| hsa-let-7g-5p | | 6839 | | | 0.71 | hsa-miR-26a-5p | | 5992 | | 0.65 |  |  |  |
| hsa-let-7a-5p | | 6710 | | | 0.70 | hsa-let-7a-5p | | 5948 | | 0.64 |  |  |  |
| hsa-let-7i-5p | | 6599 | | | 0.69 | hsa-let-7c | | 4739 | | 0.51 |  |  |  |
| hsa-miR-451a | | 6457 | | | 0.67 | hsa-miR-27b-3p | | 3625 | | 0.39 |  |  |  |
|  | |  | | |  |  | |  | |  |  |  |  |
