# Supplementary material for: High-Throughput Sequencing Approach Uncovers the miRNome of Peritoneal Endometriotic Lesions and Adjacent Healthy Tissues
Source: PLoS One. 2014 Nov 11;9(11):e112630. doi: 10.1371/journal.pone.0112630 (PMC4227690; doi:10.1371/journal.pone.0112630)
Supplement: Table S7 — The list of potential novel miRNAs proposed by miRDeep2. (DOCX) [file pone.0112630.s008.docx]

| Table S7. The list of potential novel miRNAs proposed by miRDeep2 | | | | | | | | | | | |
| --- | --- | --- | --- | --- | --- | --- | --- | --- | --- | --- | --- |
|  | **Total mature read count** | | | | | | | | | | |
|  | **Endometrium** | **Lesion** | **Healthy**  **tissue** | **Lesion** | **Healthy tissue** | **Lesion** | **Healthy tissue** | **Endometrium** | **Lesion** | **Healthy tissue** | **Lesion** |
| **precursor coordinate** | **E47.1** | **E47.3** | **E47.4** | **E47.5** | **E47.6** | **E47.8** | **E47.9** | **E101.1** | **E101.2** | **E101.3** | **E101.4** |
| chr19:58024386..58024428:- |  | 103 | 75 | 215 |  |  | 124 | 378 | 24 |  |  |
| chr19:40458803..40458848:+ | 352 |  |  |  |  |  |  | 424 |  |  |  |
| chr3:191377087..191377124:- | 129 | 29 |  |  |  | 107 |  |  |  |  |  |
| chr8:54935564..54935624:+ |  |  |  |  |  |  |  |  |  | 15 |  |
| chr5:170221993..170222048:+ |  |  |  |  |  |  |  | 12 |  |  |  |
| chr5:174790685..174790764:- |  |  |  |  |  | 1970 |  |  |  |  |  |
| chr11:62360192..62360234:+ |  |  |  |  |  | 213 |  |  |  |  |  |
| chr17:41960422..41960481:- |  |  |  |  |  |  |  |  |  | 11 |  |
