# Supplementary material for: High-Throughput Sequencing Approach Uncovers the miRNome of Peritoneal Endometriotic Lesions and Adjacent Healthy Tissues
Source: PLoS One. 2014 Nov 11;9(11):e112630. doi: 10.1371/journal.pone.0112630 (PMC4227690; doi:10.1371/journal.pone.0112630)
Supplement: Table S8 — Read counts of differentially expressed miRNAs in studied samples. (DOCX) [file pone.0112630.s009.docx]

| Table S8. Read counts of differentially expressed miRNAs in studied samples. | | | | | | | | | | | |
| --- | --- | --- | --- | --- | --- | --- | --- | --- | --- | --- | --- |
| Normalised read count (counts per million) | | | | | | | | | | | |
| **miRNA ID** | **Endometrium** | **Lesion** | **Healthy tissue** | **Lesion** | **Healthy tissue** | **Lesion** | **Healthy tissue** | **Endometrium** | **Lesion** | **Healthy tissue** | **Lesion** |
|  | **E47.1** | **E47.3** | **E47.4** | **E47.5** | **E47.6** | **E47.8** | **E47.9** | **E101.1** | **E101.2** | **E101.3** | **E101.4** |
| hsa-miR-449a | 394 | 5 | 0 | 70 | 1 | 4 | 1 | 91 | 8 | 0 | 1 |
| hsa-miR-34c-5p | 1680 | 26 | 9 | 1044 | 21 | 109 | 16 | 1692 | 59 | 2 | 33 |
| hsa-miR-200a-3p | 487 | 11 | 4 | 70 | 10 | 4 | 12 | 26 | 0 | 0 | 1 |
| hsa-miR-200b-3p | 1476 | 24 | 8 | 143 | 21 | 17 | 16 | 0 | 0 | 0 | 0 |
| hsa-miR-141-3p | 3727 | 6 | 1 | 21 | 12 | 98 | 40 | 2 | 0 | 0 | 0 |
